# Serum/Plasma Proteome in Non-Malignant Liver Disease

**DOI:** 10.3390/ijms25042008

**Published:** 2024-02-07

**Authors:** Lei Fu, Nurdan Guldiken, Katharina Remih, Anna Sophie Karl, Christian Preisinger, Pavel Strnad

**Affiliations:** 1 Department of Internal Medicine III, Gastroenterology, Metabolic Diseases and Intensive Care, University Hospital RWTH Aachen, Pauwelsstraße 30, 52074 Aachen, Germany; lfu@ukaachen.de (L.F.); ngueldiken@ukaachen.de (N.G.); kremih@ukaachen.de (K.R.); akarl@ukaachen.de (A.S.K.); 2 Proteomics Facility, Interdisciplinary Centre for Clinical Research (IZKF), Medical School, RWTH Aachen University, Pauwelsstraße 30, 52074 Aachen, Germany; cpreisinger@ukaachen.de

**Keywords:** serum/plasma proteome, biomarker, liver disease

## Abstract

The liver is the central metabolic organ and produces 85–90% of the proteins found in plasma. Accordingly, the plasma proteome is an attractive source of liver disease biomarkers that reflects the different cell types present in this organ, as well as the processes such as responses to acute and chronic injury or the formation of an extracellular matrix. In the first part, we summarize the biomarkers routinely used in clinical evaluations and their biological relevance in the different stages of non-malignant liver disease. Later, we describe the current proteomic approaches, including mass spectrometry and affinity-based techniques, that allow a more comprehensive assessment of the liver function but also require complex data processing. The many approaches of analysis and interpretation and their potential caveats are delineated. While these advances hold the promise to transform our understanding of liver diseases and support the development and validation of new liver-related drugs, an interdisciplinary collaboration is needed.

## 1. Introduction

The liver constitutes the largest gland of the human body and is a central metabolic hub. It receives nutrients from the intestine that are either stored or re-processed in the parenchymal cells, the hepatocytes, before being secreted into the bloodstream [1]. Due to that, hepatocytes are at the center of lipid, glucose and protein metabolism, and are responsible for the production of a wide range of serum proteins. They synthesize approximately 10–20 g proteins daily [2] and the plasma proteome reflects the synthetic and secretory processes in the liver. In addition to that, the intracellular proteins released into plasma are commonly assessed as so-called liver function tests (LFTs). Among them, aspartate and alanine amino transferases (ASTs/ALTs) are most widely used [3]. These are enzymes that are enriched in hepatocytes and their release into the plasma increases under stress situations as well as during cell death. Since their serum half-life is relatively short (<24 h for ASTs, ~2 d for ALTs), they mirror the recent liver challenges and may become elevated due to various stressful conditions such as viral infection or intake of hepatotoxic drugs [3]. However, life-threatening liver disorders often develop over many years, where persistent stress leads to the activation of hepatic stellate cells and increased production of extracellular matrix that results in progressive liver scarring. This process is called fibrogenesis, and while intermediate fibrosis stages are clinically inapparent, end-stage fibrosis, also termed liver cirrhosis, is associated with greatly increased liver mortality [4,5]. The latter is characterized by extensive remodeling of the liver architecture, hepatocyte loss and decreased levels of many hepatocyte-made proteins [6]. While the synthesis of hepatocellular proteins tends to reflect the amount of functioning hepatocytes, it is also the subject of various regulations such as the well-known acute phase reaction. This is triggered by cytokines that increase the production of so-called acute-phase proteins (APPs) and decrease the synthesis of anti-APPs [2]. The manufacturing of type 1 APPs (such as complement C3) is induced by interleukin 1-like cytokines, while the generation of type 2 APPs (such as hepcidin or fibrinogen) is stimulated via the interleukin 6 family of cytokines [2,7]. The classic negative APPs are albumin or transferrin [2].

While the usefulness of hepatocellular proteins in the short-term monitoring of liver stress and their changes in advanced liver fibrosis/cirrhosis are well established, their usefulness in intermediate fibrosis stages is less explored. In fact, the scores that are sometimes used in this setting often rely on a combination of LFTs with surrogates of advanced liver fibrosis with the presence of portal hypertension (such as the AST-to-platelet ratio [APRI]) or on an assessment of the proteins involved in the production/remodeling of the extracellular matrix (ECM; such as the ELF test) [8]. However, recent systematic analyses of the serum proteome suggest that many more proteins might be used in this respect and that such comprehensive analyses may result in a precise assessment of the liver status [9]. Such a non-invasive prediction would be of great relevance, since liver biopsy, the current gold standard for the evaluation of liver fibrosis, is costly, risky and often inaccurate, since it examines only a small portion of the liver [8]. As a result, the current review summarizes the usefulness of plasma/serum proteins in the assessment of liver function with a particular focus on novel proteomic techniques that have the potential to revolutionize our assessment of liver diseases. The current data suggest that proteomics is particularly useful for the non-invasive estimation of the liver fibrosis stage, while its usefulness in discriminating between liver disease etiologies needs to be further explored. While our review focuses on non-malignant liver disease, several excellent articles described proteomic changes occurring in hepatocellular carcinoma (HCC) [10,11] and some of these findings might become useful for its non-invasive detection [12,13]. The review starts with a description of the traditional biomarkers/candidates (Section 2) and continues with a description of proteomic techniques (Section 3) and the methods for their analysis (Section 4).

## 2. Overview of Traditional Protein Biomarkers and Their Usefulness

**Figure 1:**
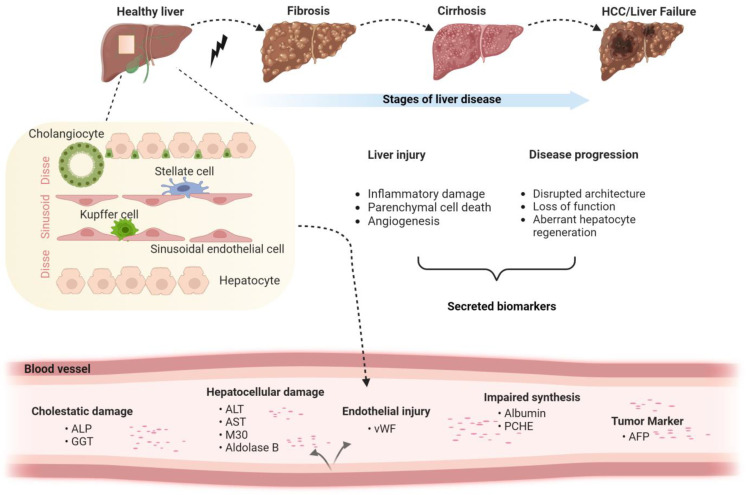
Liver disease stages and corresponding cell type-specific biomarkers. The schematic depicts the stages of liver disease, the major hepatic cell types and the corresponding biomarkers with a focus on non-malignant disorders (Created with BioRender.com).

As mentioned above, multiple biomarkers were suggested to mirror different aspects of liver disease (Figure 1) ([3,8]; for further details see below). Many of them are related to hepatocytes, the parenchymal cells of the liver that make up 80% of the liver volume [14]. These can be subdivided into “leakage markers” (i.e., proteins that are typically found intracellularly but are released into the bloodstream during liver injury) such as AST or ALT; differentiation markers (i.e., proteins that are produced in primarily in less differentiated hepatocytes) such as alpha-fetoprotein (AFP); and synthesis markers such as albumin or transferrin (Table 1) [15,16,17,18]. Other proteins expressed either by stressed hepatocyte or by inflammatory cells, such as CXCL10 or C-reactive protein (CRP), also mirror the extent of hepatic inflammation/injury. Fibrogenesis can be evaluated directly using the level of ECM components (i.e., collagen or collagen-cleavage products such as pro-C3, hyaluronic acid, etc.) and/or levels of proteins involved in ECM production/degradation such as matrix metalloproteinases (MMPs) and tissue inhibitors of metalloproteinases (TIMPs) [8]. Finally, advanced liver fibrosis is characterized by extensive vascular remodeling [19], affecting endothelial cell types and their products such as von Willebrand factor (vWF) [19,20,21]. In the following chapters, we will describe an array of protein biomarkers and discuss both their advantages and disadvantages.

### 2.1. Liver Injury Markers

Cell death in the liver is triggered by numerous hepatotoxic factors such as metabolic disorders, alcohol consumption, drug intoxication and microbial infections [26,27]. It leads to the release of intracellular molecules of proteins, protein fragments and microRNAs into the circulation [2,26,27]. Extracellular vesicles also constitute attractive biomarkers and their potential usefulness has been addressed by several recent reviews [28].

#### 2.1.1. Aspartate/Alanine Amino Transferase

AST and ALT are well-known “leakage markers” reflecting the extent of hepatocellular injury [29]. ALT is more hepatocyte-specific and has a longer serum half-life than AST [30]. The AST/ALT ratio can be used to distinguish between different injury types. AST constitutes a mitochondrial enzyme that is released in a more severe injury, while ALT is cytoplasmic. An AST/ALT ratio of over 2 is suggestive of alcoholic liver disease, but an increased AST/ALT ratio (i.e., >1) is also seen in advanced liver fibrosis that is characterized by the loss of hepatocytes [31,32]. The fact that AST remains elevated in advanced liver fibrosis led to its incorporation into several non-invasive fibrosis scores such as the APRI and the fibrosis-4 index (FIB-4) [8,33]. In both cases, AST is divided by the platelet count, since the platelet count decreases in advanced liver fibrosis due to the presence of portal hypertension [34]. Notably, a combination of several liver-related parameters (i.e., AST and ALT as used in FIB-4 or even more variables as employed in the LiverRisk score) might be superior to the simpler formula [35]. Moreover, gamma-glutamyl transferase (GGT) is a potentially useful adjunct for such scores since it is an established marker of liver steatosis [36], which is an important constituent of the most prevalent liver disorders, i.e., alcoholic and non-alcoholic fatty liver disease [37]. However, it needs to be kept in mind that these biomarkers per se are unrelated to the fibrosis process and because of that a combination with ECM-related markers should be considered to improve the detection of fibrosis stages.

#### 2.1.2. Soluble Keratin 18 (K18) and Fragmented K18

Keratin 18 (K18) is an abundant intermediate filament protein of most single-layered and glandular epithelia [38]. It constitutes an intracellular protein that is released into the blood during cell injury [27,39]. K18 is cleaved by caspases resulting in 30 kDa and 45 kDa fragments [40]. The former can be detected by the so-called M30 ELISA, while M65 measures a broader range of K18 products and therefore represents an etiology-unspecific marker of epithelial injury [27,39,41]. The ratio of M65:M30 can be used as a marker of different types of hepatocyte death, while the ratio of M65: ALT might be used to distinguish patients with acute alcoholic hepatitis from patients with non-alcoholic steatohepatitis [42]. 

The usefulness of K18-based biomarkers was particularly well evaluated in the non-alcoholic and alcoholic fatty liver disease, and several interesting findings suggest that it might be useful in the clinical routine: (i) it seems to be more sensitive to mild inflammation and might therefore be useful to differentiate steatohepatitis from simple steatosis [43,44]; (ii) it mirrors the extent of intrahepatic inflammation, which might be particularly useful to differentiate alcoholic hepatitis from alcoholic liver disease [42,45,46]. Due to its ability to faithfully reflect the extent of hepatic inflammation, M30 might be useful in identifying the subset of subjects with severe alcoholic hepatitis that benefit from prednisolone treatment [45]. However, it needs to be kept in mind that K18 is expressed in a large range of epithelial tissues and because of that is elevated in multiple other diseases (chronic lung allograft dysfunction, COVID-19, non-small cell lung cancer, intestinal graft-versus-host disease, etc.) [39]. In line with that, the tissue polypeptide-specific antigen that recognizes K18 is a well-known tumor marker, while tissue polypeptide antigen is another tumor marker and the corresponding assay detects various keratins including K18 [39].

#### 2.1.3. Aldolase B

Aldolase B (ALDOB) is another potential marker of hepatocellular injury, although it is also found in kidney and intestinal epithelial cells [47,48]. It is considered a good indicator of liver cell necrosis given that serum ALDOB levels are increased in patients with acute and chronic hepatitis [48]. Mass spectrometry (MS)-based proteomic studies demonstrated that ALDOB levels moderately correlate with both ALT (r = 0.391; *p* = 0.001) and AST (r = 0.523; *p* < 0.001), and are more elevated in patients with hepatocellular DILI (drug-induced liver injury) compared to cholestatic DILI. In a cohort consisting of 137 controls and 459 individuals with ALD assessed with MS-based proteomics, ALDOB correlated well with both fibrosis stage and steatosis grade [9]. In a study based on targeted MS serum proteomics involving 133 DILI patients, ALDOB (r = 0.90) demonstrated a stronger correlation with ALT than CK18, suggesting that ALDOB is highly liver-specific. With regard to liver zonation, spatial transcriptomics demonstrated that ALDOB is enriched in mid-lobule hepatocytes [49]. Therefore, ALDOB can be combined with other hepatocellular biomarkers to assess the most affected parts of the liver lobe.

#### 2.1.4. Golgi Protein 73 (GP73)

GP73, also known as GOLPH2 and GOLM1, represents a 73-kDa resident Golgi type II transmembrane glycoprotein expressed in various epithelial cells [50,51]. In healthy livers, GP73 is produced in biliary epithelia and only minimally in hepatocytes. However, its hepatocellular expression is upregulated in acute liver injury as well as in advanced liver fibrosis [50,52]. In compensated cirrhosis, GP73 levels positively correlate with the amount of portal hypertension [53]. GP73 also serves as a serum marker for hepatocellular carcinoma [51,54] and its silencing decreases the invasiveness of this tumor [55]. These findings underscore that GP73 is not only a biomarker, but may also be involved in disease pathogenesis.

### 2.2. Biomarkers of Advanced Liver Disease

#### 2.2.1. Von Willebrand Factor (vWF)

VWF is a glycoprotein synthesized primarily by endothelial cells, whose secretion is promoted by inflammatory mediators as well as endothelial damage [56]. It supports platelet adhesion and because of this it might functionally compensate for the decreased platelet levels in subjects with cirrhosis [57]. Similarly, highly elevated vWF levels were detected in subjects with acute liver failure (ALF) and were again suggested to support platelet function, despite its relative loss of function [58].

The above-described observations led to the assessment of vWF levels in multiple cohorts of patients with advanced liver fibrosis. It has been shown that vWF correlates with the extent of portal hypertension [59] and because of that it gradually rises in subjects with compensated cirrhosis and even more in decompensated cirrhosis [60]. Therefore, it is not surprising that elevated vWF levels are associated with an increased risk of decompensation and liver-related mortality [60], and were suggested as a valuable adjunct to the predictive scores [61,62].

#### 2.2.2. Apolipoproteins

Apolipoproteins are components of plasma lipoproteins and are mainly synthesized in the small intestine and liver [63,64,65]. Their secretion is affected by multiple players such as nutritive status or cytokines [66,67]. Given the key importance of the liver for lipid metabolism, it is not surprising that advanced liver disease is paralleled by alterations in the lipoprotein composition. This is particularly true for the so-called high-density lipoproteins (HDLs) that serve as inhibitors of inflammatory responses [68] and restrained liver injury in an experimental model [69]. In line with that, decreased levels of HDL cholesterol and apolipoproteins A1 (APOA1) were important predictors of poor survival in subjects with compensated/decompensated liver cirrhosis [70]. The prognostic relevance of HDL-cholesterol as well as APOA1 was confirmed in independent cohorts [6,71]. However, the alterations in apolipoprotein composition extend well beyond HDL. In particular, a proteomic analysis revealed that APOA1, APOA2, APOB, APOC1, APOC3, APOC4, APOF, APOH, APOL1 and APOM correlate with severity of liver fibrosis [9]; however, different apolipoproteins show different patterns. For example, increased APOA2 levels were correlated with higher steatosis stages, while decreased APOF levels were observed in more advanced fibrosis stages [9]. ApoB was suggested as a biomarker of steatosis, while APOE was associated with NASH [72]. Therefore, while apolipoproteins constitute attractive biomarkers reflecting liver status, further studies are needed to fully explore their usefulness.

#### 2.2.3. Pseudocholinesterase (PCHE)

PCHE, also known as plasma/serum cholinesterase (ChE), acetylcholine acetylhydrolase and butyrylcholinesterase (BuChE, BCHE), is produced by hepatocytes [73]. Its levels decrease in acute and chronic liver diseases [74]. Although the serum PCHE activity decreased in advanced liver disorders [6,9,74], some reports claimed increased levels in compensated cirrhosis [75]. Lower levels of plasma PCHE were associated with the presence of significant liver fibrosis and hepatic inflammation (vs. no/minimal fibrosis/inflammation), but did not correlate with the degree of hepatic steatosis [9]. PCHE was found as the most downregulated protein when cirrhotics were compared with healthy subjects, which might be both due to the loss of hepatocytes and the concomitant inflammatory reaction [6,76]. Despite that, serum BCHE levels were only relatively poor predictors of 90-day mortality in decompensated cirrhotics (AUROC 0.63) [6].

## 3. Proteomics as the Next Generation Approach

### 3.1. Key Methods

The standard method for the discovery of novel protein biomarkers is the non-targeted analysis of proteins in serum through the use of mass spectrometry (MS)-based proteomics [77]. This approach is also used for investigating protein differences in tissues, cells or any other form of protein-containing entities. Most commonly, purified serum or plasma is digested with a specific protease—usually trypsin—and the resulting peptide mixture is subjected to mass spectrometry coupled to a nano-flow liquid chromatography (LC) system [78]. The raw data are subsequently analyzed using respective software packages against the corresponding human database followed by downstream bioinformatic workflows including the identification of the corresponding proteins from the analyzed peptides. This approach, termed “bottom–up shotgun proteomics” [79], has been in use since the mid-1990s, where “bottom–up” refers to the identification (and quantification) of proteins from the detected individual peptides and the term “shotgun” reflects the non-targeted nature of the investigational approach [79]. Back then, proteins were separated using two-dimensional (2D) gel electrophoresis, and spots of interest were excised, manually digested and analyzed using matrix-assisted laser desorption and ionization (MALDI)- or LC-coupled ESI-based mass spectrometers [80]. This was very tedious and time consuming, and only allowed for limited identification rates; the major limitations were reproducibility issues and a very high workload. The introduction of Orbitrap generation instruments (2005, Thermo Scientific) [81], as well as more sophisticated time-of-flight instruments (such as the Impact (Bruker) or TripleTOF Systems (Sciex)) [82,83], allowed for analysis of more samples in a shorter time with higher mass accuracy and resolution. Further technical developments in the last decade have now resulted in the possibility of performing true and robust high throughput analysis of hundreds of serum samples within reasonably short time frames. These advancements include (a) the routine implementation of ion mobility as an additional level of peptide separation in proteomics (e.g., timsTOF instruments from Bruker [84] or the FAIMS unit on Thermo instruments [85,86]); (b) further instrumental development by MS manufacturers, drastically increasing scanning speed, mass accuracy and resolution; (c) the development and implementation of chromatographic materials, methods and systems [87], enabling very short gradients and thus increasing the maximum number of “samples per day” (SPD) that can be analyzed. In previous years, it was common to use 30 or 60 min gradients for semi-complex samples, which can now be lowered to 5–10 min gradient length, thus increasing the number of SPD [85]. A further aspect of utmost importance is the recent development, improvement and implementation of “data-independent acquisition” (DIA) methods [88,89]. Data dependent acquisition (DDA) methods have been the method of choice in the proteomic field since the late 1990s. The underlying principle with DDA is that after every MS1 overview scan, only a limited number of peptides/analytes are fragmented (MS2 spectra)—usually the 5 to 20 most intense peaks [78,79]. This approach allows for an extremely sound and robust identification of the higher concentrated proteins in a sample, but will always miss out on less abundant proteins. This shortfall could be overcome with DIA-based MS, which has gained significant momentum over the last decade [90]. DIA utilizes, all masses detected in an overview scan, which are then subsequently fragmented. This allows for a higher coverage of peptides (and therefore proteins) in any given sample. These approaches (including the “sequential window acquisition of all theoretical mass spectra” (SWATH-MS) approach [83,91]) have recently been implemented into routine use [9], also as a consequence of the development of novel bioinformatic tools such as DIA-NN [92,93] and MSFragger-DIA [94], reducing the burden of the computational workload and complexity of these analyses. 

While the above text describes the discovery-driven method of identifying novel biomarkers from serum/plasma in various disease using a non-targeted approach, targeted analysis using dedicated MS methods are in use for the validation of these findings in larger cohorts. These techniques involve at first the selection of appropriate candidates, a thorough analysis of the physiochemical properties and suitability of individual peptide sequences. Subsequently, labelled peptides are synthesized and single-/multi-/parallel-reaction monitoring (SRM, MRM, PRM) methods are established [95]. This approach facilitates and enables an in-depth investigation of prospective biomarkers in a large-scale validation cohort (see peptideatlas.org and srmatlas.org) [96]. 

#### 3.1.1. Non-Depleted vs. Depleted Proteomics

While it is nowadays a routine approach to identify and quantify >8000 proteins in tissue or cell lysates in a single-shot bottom–up MS experiment using a feasibly short LC gradient with the approaches described above, this is not possible when analyzing serum or plasma. This is due to the enormous differences in protein concentrations in these body fluids [97]. The range of protein concentrations spans a range of 10–12 orders of magnitude (from low pg/high fg/mL to approximately 40 mg/mL for albumin) leading to the major caveat in serum proteome analysis that only 24 extremely highly concentrated proteins contribute to 99% of the entire protein amount in serum or plasma (see [98,99]). Due to these limitations, it is only possible to reproducibly identify, quantify and analyze several hundreds of proteins in non-depleted serum and plasma within a reasonable amount of time. This may be sufficient for questions aimed at pathologically relevant changes of proteins in the upper concentration range, but most likely will only be suitable for a minority of studies. For instance, when analyzing a hepatocellular malfunction it can be of major interest to focus on the abundant serum proteins that originate from the liver [6]. Most other work will require an in-depth analysis of proteins with significantly lower concentrations. One common approach to reduce the complexity is using fractionation of the serum samples, either at the protein or the peptide level. This can be achieved through protein separation using standard techniques, such as 1/2-dimensional gel electrophoresis, or classic chromatography approaches, such as anion and/or cation exchange chromatography. Peptide fractionation may be achieved using methods such as (high pH) reversed-phase chromatography and strong cation or strong/weak anion exchange in order to reduce the enormous complexity of the peptide mixture derived from the proteolytical digest [100]. These approaches aid in increasing the depth of low-abundant serum protein quantitation but come at the cost of severely elongated MS run time, making it unfeasible for large-scale high-throughput analysis.

Another very common approach to enable a more in-depth analysis of serum proteins of lower concentrations is the depletion of the most abundant serum/plasma proteins. Several vendors provide reagents ranging from depleting only albumin (and immunoglobulins) to the depletion of 14 highly abundant proteins (albumin, alpha-1-acid glycoprotein, alpha-1-antitrypsin, alpha-2-macroglobulin, apolipoprotein A-I, fibrinogen, haptoglobin, IgA, IgG, IgM and transferrin (both depleted using Agilent Multiple Affinity Removal Column Human 14 and Thermo High-Select™ Top14 Abundant Protein Depletion Resin) with the addition of apolipoprotein A-II, complement C3 and transthyretin (Agilent) or IgD, IgE and light chain IgG (Thermo)). Furthermore, enrichment methods such as the Proteominer approach (BioRad) as well as small molecule-based probes (such as ATP or cAMP) have also been employed [101]. The depletion approach, despite having obvious advantages, has also received criticism for potentially depleting less abundant proteins of interest due to their potential interactions with albumin or others (discussed in [99]). An interesting approach was the combination of depleted plasma and high pH reverse fractionation of the digested peptides to generate an extremely comprehensive plasma protein library, followed by the analysis of non-depleted patient samples [102]. Another promising, rather new technology, is the analysis of the protein corona on diversely modified magnetic nanoparticles, which have been successfully used for the in-depth investigation of plasma proteins using DIA-based MS analysis [103,104].

Other biochemical methods aiming at targeted analysis include ELISA tests, Western blotting and protein arrays. While the former relies on the use of specific primary antibodies against the protein(s) of interest, the latter comprises an analysis method with the possibility to screen for dozens of proteins at the same time [105]. 

#### 3.1.2. Mass Spec. vs. Affinity-Based Methods (Olink and Somascan) 

The continued development of affinity-based assays for multiplexed protein identification has helped in establishing these techniques for use in high-throughput serum/plasma proteomic studies. A number of companies have made these plate-based assays widely available, covering different depths of potential biomarkers. The most prominent techniques in this field are the proximity extension assay (Olink Bioscience, Uppsala, Sweden) and the aptamer-based SomaScan technology (SomaLogic, Boulder, CO, USA). While the former is constructed on paired bodies that are linked to complementary oligonucleotide sequences using quantitative polymerase chain reaction or next-generation sequencing as readout [106,107], the latter employs a library of highly specific DNA aptamers that are linked to fluorophores that provide the quantification signal [108,109].

Both assays only require a small amount of sample volume and are able to capture a large number of proteins across the dynamic range of human serum/plasma, and, in contrast to mass spectrometry, are less affected by it. However, since they only provide indirect measurements of their target proteins, the specificity and accuracy might be affected by, e.g., post-translational modification or off-target binding. Several studies have compared both approaches and the correlation among their readouts [110,111,112]. Due to differences in protein detection, targeted epitopes or underlying quantification techniques, among others, correlations varied widely, from excellent concordance in a small number of features to very low correlation in a substantial number of cases. These discrepancies complicate the adoption of these techniques into clinical practice where accurate identification and quantification are crucial. Notably, a poor correlation across assays is certainly not a unique phenomenon to the novel platforms, as different commercially available immunoassays targeting multiple proteins often show poor inter-assay correlations as well [113].

## 4. Data Analysis of Serum Proteomics 

### 4.1. Bioinformatic Methods

Despite the technological progress made over recent years, proteomic data are still susceptible to variations caused by non-biological sources (systematic bias, e.g., introduced by differences in sample preparation/handling, device calibration, etc.). Therefore, normalization is employed to minimize this bias and to produce comparable and reliable results. Several methods have been developed and are based on different statistical assumptions. Since the distribution of protein abundances is often highly skewed, a logarithmic transformation is usually applied before the normalization step. While several normalization techniques are currently used, Välikangas et al. systematically compared different approaches and concluded that the majority of them yield a similar performance in most proteomic datasets [114].

Another caveat is that the raw quantitative data often contain high numbers of missing values (MVs), which can be due to both technical and biological reasons, such as ion competition, poor ionization efficiency, incomplete protein digestion or low expression levels. When all MVs are removed or the data are left as they are, the following statistical analyses are drastically skewed, and incorrect conclusions may result [115]. Alternatively, these missing values can be accounted for with different imputation techniques that take the distribution of detected proteins into consideration. Several imputation algorithms and machine learning models have been developed. The most suitable technique strongly depends on the overall nature/origin of the MVs and many investigations have been performed to support the decision-making process [116].

Moreover, the optimal pre-processing methods depend on the experimental environment or the underlying research question. Visualization techniques such as hierarchical clustering or heatmaps can aid in determining the usefulness of the applied steps. Once the data are readily processed, they are subjected to further statistical and downstream analyses.

A relatively straightforward approach is the comparison of mean protein abundances between the groups of interest. Traditional techniques such as t-tests or ANOVA can be applied, but especially in cases of small sample sizes, the statistical power of these tests might be impaired, resulting in insignificant p-values and/or a large variance. To address this issue, several statistical models have been proposed, such as the moderated t-statistics from the empirical Bayes procedure Linear Models for Microarray Data (LIMMA) [117], other linear mixed-effect models [118], mean/median sweeps [119] and “masterpool” normalization [118]. Since these models simultaneously test multiple hypotheses in a high-dimensional dataset, it is crucial to set up an appropriate false discovery rate threshold to reduce the number of false positive results. Commonly used methods are the Benjamini–Hochberg procedure and FDR estimation from permutations [120].

**Figure 2:**
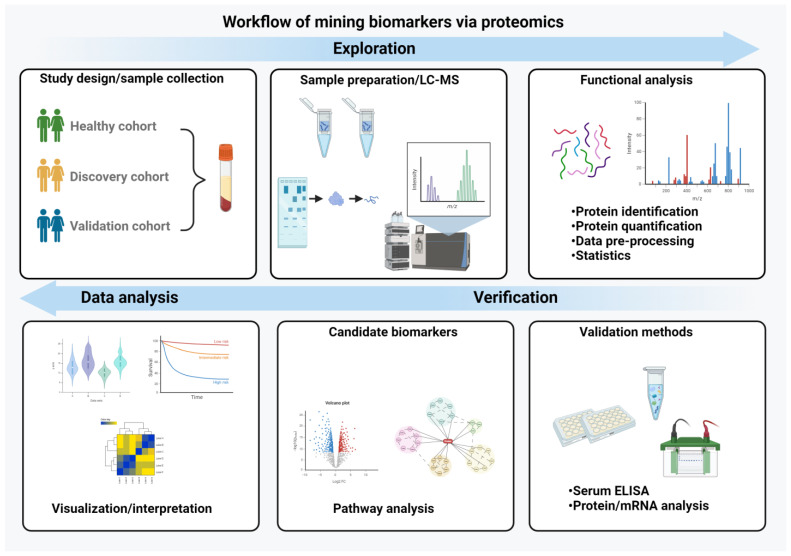
Workflow of biomarker mining. The essential steps in the biomarker discovery process are depicted. It begins with study design and a careful sample preparation and includes a functional analysis, validation of the data with independent methods and their interpretation and visualization. (Created with BioRender.com)

Finally, the observed proteomic changes need to be placed into a biological context. For example, over-representation analysis (ORA) assesses whether specific biological categories (such as Gene Ontology [GO] terms, pathways or protein complexes) are over-represented among the altered proteins [121]. While ORA focuses on identifying over-represented categories only, functional enrichment analysis also assesses an overall function profile. Protein set enrichment analysis (PSEA) considers the enrichment of functional annotations across a ranked list of proteins, which is based on expression values or other defined metrics [122]. Given that proteins typically function in a transient or stable complex with other proteins, interaction databases (e.g., STRING, MINT or BioGRID) [123,124,125] are employed to gain further insights into their biological function (Figure 2).

### 4.2. Key Papers

Even though the liver produces the 85–90% of serum/plasma proteins [126] and liver diseases are therefore an obvious target for biomarker research, the field was surprisingly silent for many years. A likely reason for this is the fact that liver-related death is relatively infrequent and advanced liver disease develops for many years, which makes it challenging to put together cohorts with a sufficient number of hard endpoints. As a result, many studies focused on biomarkers reflecting the histological features rather than predicting liver-related death. In a milestone paper, Niu et al. used a combination of liver and plasma proteomics and demonstrated that the latter were superior to routinely used biomarkers in predicting not only histological inflammation and fibrosis, but also future liver-related events and all-cause mortality [9]. While the authors have to be commended for their pioneering effort, their results rely on a state-of-the art technology that is not widely available. The SOMAscan technique was also tested in different liver disease cohorts and yielded interesting mechanistic insights [127,128,129]. It might be useful to reliably identify subjects with risky non-alcoholic steatohepatitis [130]. With regard to the latter, an eight-protein biomarker panel was superior to the currently used surrogates and ADAMTSL2 emerged as a novel, attractive biomarker [130]. Finally, a recent manuscript applied the Olink platform to a large population-based cohort and identified several potential biomarkers of fatty liver disease as well as reflecting liver fibrosis [131].

## 5. Conclusions and Future Directions

While hepatologic research was long hampered by a lack of large, robust cohorts with long-term follow-ups, such cohorts are now available, and this has led to breakthroughs and genetic discoveries. Subsequently, these cohorts were used for transcriptomic analyses and are now increasingly explored for metabolomic and proteomic studies. The latter efforts are emboldened by an increased availability of high-throughput affinity-based proteomic platforms, as well as computational innovations such as artificial intelligence. Collectively, these developments will likely lead to an expansion in large-scale proteomic studies and yield novel, attractive disease biomarkers. An example of potentially attractive biomarkers emerging from independent proteomic studies include ADAMTSL2 and the members of the aldo-keto reductase family [9,129,130]. While the translation of such achievements into the clinical routine is often challenging and requires a validation in large prospective trials, the current surge in liver-related clinical trials may facilitate this process. To that end, proteomic-based panels may not only help to identify subjects that are at risk for a more aggressive liver disease, but also disease subgroups that respond well to a particular treatment. At the same time, it has to be kept in mind that proteomic techniques are not widely available and might be too laborious for high-throughput diagnosis. Consequently, the identification of novel biomarkers needs to be coupled with efforts facilitating their widespread use such as the development of robust immunoassays. 

## Figures and Tables

**Table 1:** Overview of widely used biomarkers of non-malignant liver disease.

Biomarkers	MW (kDa)	Normal Serum Values *	Pros	Cons
Hepatocyte injury markers				
M30	30	48.3 to 217.1 U/L ^a^	Sensitive to hepatocellular injury	Not widely available
ALT	54.6	10 to 40 U/L	Widely available	Less useful in advanced liver disease
AST	46.1	10 to 59 U/L	Widely available	Not very specific
Aldolase B	160	1.0 to 7.5 U/L	Liver-specific	Not widely available
Secreted hepatocellular proteins				
PCHE	342	4.9 to 11.9 U/mL	Widely available	False low in some patients
ALB	66.5	32 to 52 g/L	Widely available	Long half-life, non-specific
Apolipoprotein A-1	28.96	0.94–1.99 g/L		Not widely available
Vn	75	239 to 711 mg/L ^b^		Not widely available
SAA	11.4 to 12.5	20 mg/mL ^c^		Not widely available
Other				
AFP	~70	<15 ng/mL	Widely available	Some tumors are AFP-negative
vWF	500–20,000	50 to 200 IU/dL ^d^	Widely available	Complex biology

* Unless otherwise stated, data were obtained from the “Clinical chemistry laboratory: Reference range values in clinical chemistry, Professional services manual.” Baltimore, Department of Pathology, University of Maryland Medical System. ^a^ The reference range for serum M30 was based on a study with a large number of healthy controls [22]. ^b^ Reference ranges of serum Vitronectin concentration were based on a cohort study of healthy subjects [23]. ^c^ The average concentration was measured from 302 healthy individuals ranging in age from 21 to 100 years [24]. ^d^ Tests were performed under static conditions [25]. M30: keratin 18 fragment; ALT: Alanine aminotransferase; AST: Aminotransferase; PCHE: Pseudocholinesterase; ALB: Albumin; Vn: Vitronectin; SAA: Serum amyloid A; AFP: α-Fetoprotein; vWF: von Willebrand factor; MW: molecular weight.

## Data Availability

Not applicable.
